# A Computational Framework to Infer Human Disease-Associated Long Noncoding RNAs

**DOI:** 10.1371/journal.pone.0084408

**Published:** 2014-01-02

**Authors:** Ming-Xi Liu, Xing Chen, Geng Chen, Qing-Hua Cui, Gui-Ying Yan

**Affiliations:** 1 Academy of Mathematics and Systems Science, Chinese Academy of Sciences, Beijing, China; 2 University of Chinese Academy of Sciences, Beijing, China; 3 National Center for Mathematics and Interdisciplinary Sciences, Chinese Academy of Sciences, Beijing, China; 4 Department of Biomedical Informatics, School of Basic Medical Sciences, Peking University, Beijing, China; Harbin Institute of Technology, China

## Abstract

As a major class of noncoding RNAs, long noncoding RNAs (lncRNAs) have been implicated in various critical biological processes. Accumulating researches have linked dysregulations and mutations of lncRNAs to a variety of human disorders and diseases. However, to date, only a few human lncRNAs have been associated with diseases. Therefore, it is very important to develop a computational method to globally predict potential associated diseases for human lncRNAs. In this paper, we developed a computational framework to accomplish this by combining human lncRNA expression profiles, gene expression profiles, and human disease-associated gene data. Applying this framework to available human long intergenic noncoding RNAs (lincRNAs) expression data, we showed that the framework has reliable accuracy. As a result, for non-tissue-specific lincRNAs, the AUC of our algorithm is 0.7645, and the prediction accuracy is about 89%. This study will be helpful for identifying novel lncRNAs for human diseases, which will help in understanding the roles of lncRNAs in human diseases and facilitate treatment. The corresponding codes for our method and the predicted results are all available at http://asdcd.amss.ac.cn/MingXiLiu/lncRNA-disease.html.

## Introduction

In recent years, accumulated studies have shown that protein-coding genes account for a very small part of the mammalian whole genome, approximately 2% [1]–[8]. This fact challenges the traditional view that RNA is just an intermediary between gene and protein. Moreover, it has become increasingly apparent that the non-protein-coding portion of the genome has essential and crucial regulatory functions, even though it does not encode proteins [9]. Notably, compared with short noncoding RNAs (ncRNAs), such as microRNAs (miRNAs) or piwi-interactingRNA (piRNAs), a number of lncRNAs make up the largest proportion of ncRNAs. Usually, lncRNA is defined as an RNA molecule longer than 200 nucleotides that cannot translate to a protein [10], [11].

With the development of both experimental technology and computational methods, an increasing number of lncRNAs have been identified in the human transcriptome [12]. Furthermore, lncRNAs have been shown to play key roles in various biological processes, such as imprinting control, epigenetic regulation, cell cycle control, nuclear and cytoplasmic trafficking, differentiation, immune responses and chromosome dynamics [11], [13], [14]. Therefore, it is not surprising that dysregulations and mutations of lncRNAs have been implicated in a variety of human diseases. So far, more than 150 human diseases are associated with lncRNAs, according to the LncRNADisease database [15], such as breast cancer [16], [17], leukemia [18], [19], colon cancer [20], prostate cancer [21], Alzheimer’s disease [22], and psoriasis [23].

More and more evidences show that lncRNAs could be both a potential biomarker of human disease and a potential drug target in drug discovery and clinical treatment. For this reason, identification of potential lncRNA-associated diseases is of great importance and urgently needed. However, compared with research dedicated to disease-related gene identification [24]–[29] and disease-related miRNA prediction [30]–[33], comparatively little is currently known about lncRNAs, especially lncRNA-associated diseases. Therefore, developing a novel computational method in the absence of known lncRNA-associated diseases would be very desirable. Fortunately, research on disease-associated genes has generated a large amount of information that virtually guarantees relatively high accuracy when coupled with the development of experimental and computational methods. To solve the above problem, we first constructed the relationship between lncRNAs and genes based on their expression profiles and then identified potential relationships between lncRNAs and diseases utilizing known disease-associated genes.

To evaluate the performance of our method, we implemented case studies and cross validation based on known experimentally verified lncRNA-disease associations from the LncRNADisease database [15]. Consequently, we obtained reliable predictive accuracy. Case studies for tissue-specific lincRNAs show good performance, in which nineteen of 100 most probable lincRNA-disease associations were verified by related research conclusions. For non-tissue-specific lincRNAs, the AUC of our algorithm is 0.7645, and the prediction accuracy is about 89%.

## Materials and Methods

### Materials

In this paper, we integrated the following three kinds of datasets to construct the computational framework aiming to infer the diseases associated with human lncRNAs: lncRNA expression profiles, gene expression profiles, and human gene-disease associations, respectively. Here a brief description was given.

1. Long intergenic noncoding RNA expression profiles

Generally speaking, lncRNAs can be classified based on their position relative to protein-coding genes, including intergenic, intragenic and antisense, respectively [7], [10]. Based on our computational framework, we would utilize the expression levels of lncRNAs in several human tissue or cell types. However, at present, comprehensive expression data of lncRNAs is still unavailable. Long intergenic noncoding RNAs (lincRNAs) is a newly discovered and important type of lncRNAs, which accounts for a large fraction of the whole lncRNA set [34]. Fortunately, the expression profiles of lincRNAs could be obtained. Thus, in this paper, we used existing data of lincRNAs to implement the whole computational framework. Although we utilized expression data of lincRNAs, our computational framework could be applied to all classes of lncRNAs. First, we downloaded expression profiles of human lincRNAs in 22 human tissue or cell types from the UCSC Table Browser [35], [36], which provides the latest expression data generated through RNA-Seq technology (GRCh37/hg19; http://genome.ucsc.edu/). For a given lincRNA in a given human tissue or cell type, comprehensive information, such as its name, chromosome location, starting point in chromosome, end point in chromosome and the score corresponding to its expression level, is listed in detail. These data were then integrated, finally arriving at expression profiles of 21626 human lincRNAs in 22 human tissue or cell types (Table S1).

1. Gene expression profiles

To construct the relationships between human lincRNAs and human genes, we downloaded expression profiles of 17080 genes in 73 human tissue or cell types, based on the research of Su et al. [37]. This data of 17080 genes has already been rigorously processed and released during their work, where there is an Entrez gene ID corresponding to each gene [37].

1. Human gene-disease associations

We extracted human gene-disease associations from the DisGeNET database [38]. DisGeNET is a new gene-disease database which integrates gene-associated diseases from several public, expert, and curated data sources, as well as text mining-derived associations. This database contains relationships between human genes and several different kinds of human diseases, including Mendelian, complex and environmental diseases [38], [39]. Its data sources contain several widely used databases, such as Uniprot [40], CTD [41], GAD [42], MGD [43] and LHGDN (http://linkedlifedata.com/sources). The number of separate gene-associated diseases for each data source was shown in Table 1. Moreover, CTD contains the vast majority of gene-associated diseases in OMIM [44], which is another classical and commonly used highly credible data source. Obviously, the gene-associated disease data proposed by this database are very comprehensive for our further study. The present version of DisGeNET records 100729 associations between 9313 genes and 5286 diseases. In our work, we only collected expression profiles for 17080 genes. Using this as our baseline number, we finally selected 51762 associations between 7303 genes and 5150 diseases (Table S5). Thus, for each human disease, we obtained a set of genes which had been experimentally validated to be associated with a given disease.

**Table 1:** Number of genes, diseases and gene-associated diseases from five different data sources.

	Number of genes	Number of diseases	Number of gene-disease associations
**Uniprot**	1754	2243	2525
**CTD**	6065	4403	16382
**GAD**	2461	1395	12798
**MGD**	1253	1016	1749
**LHGDN**	6140	1847	59274

## Methods

1. Basis of the computational framework

**Figure 1:**
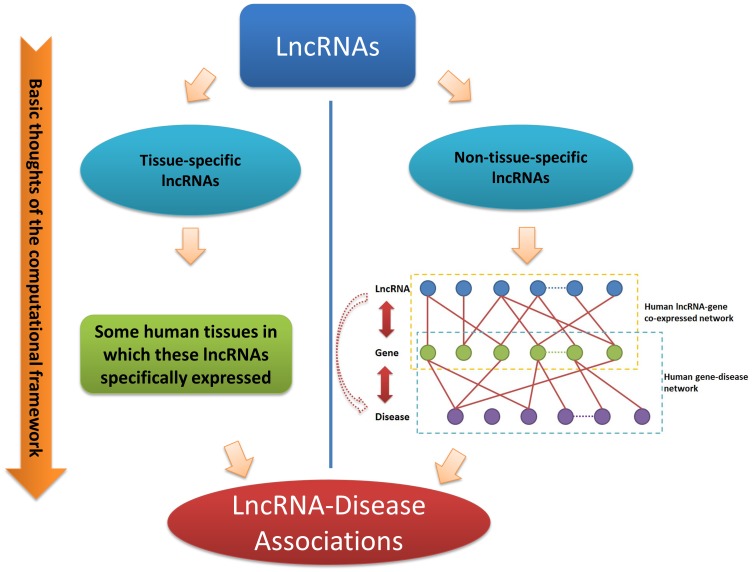
Working principles of the computational framework. Briefly, if a given lncRNA could be specifically linked with some human tissues, then we could, in turn, link it to diseases known to be related to these human tissues. Moreover, if we could find the effective associations of other lncRNAs with human genes, then we could construct a human lncRNA-gene co-expressed network and human gene-disease network and then infer the associations between lncRNAs and disease through incorporating the information provided by these two networks.

**Figure 2:**
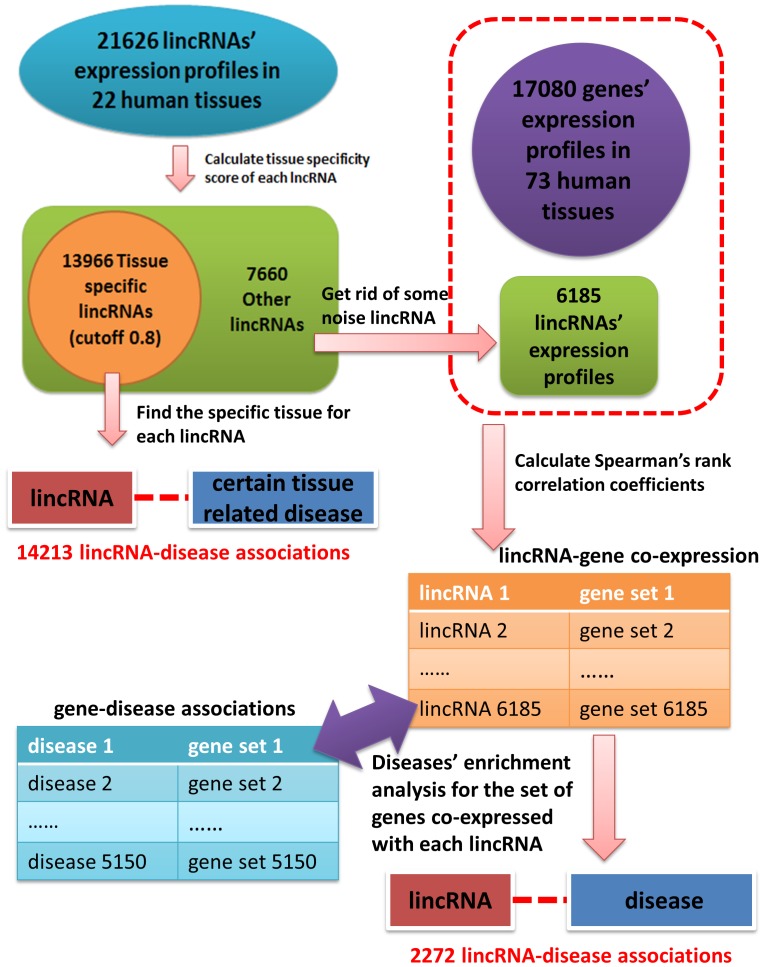
The flowchart of our method. There are four steps: (1) Calculation of tissue specificity score and partitioning all the lncRNAs to those that are tissue-specific and non-tissue-specific. (2) Prediction of potential lncRNA-associated diseases for tissue-specific lncRNAs. (3) For each non-tissue-specific lncRNA, find the corresponding genes co-expressed with this certain lncRNA through computing Spearman’s correlation coefficients. (4) Perform disease enrichment analysis for the set of genes co-expressed with each lncRNA and predict potential lncRNA-associated diseases for non-tissue-specific lncRNAs.

Based on the data collected, including the expression of human lncRNAs, human gene expression, and gene-associated diseases, we constructed a computational framework to infer diseases associated with human lncRNAs. The following logic supports this framework, as shown in Figure 1. If a given lncRNA could be specifically linked with some human tissues, then we could, in turn, link this lncRNA to diseases known to be related to these human tissues. However, for other non-tissue-specific lncRNAs, if we could find the effective associations of them with human genes, then we could utilize the known relationships between human genes and diseases and link these lncRNAs to specific human diseases. The workflow of our computational framework was shown in Figure 2.

1. Prediction of human disease-associated lncRNAs

As shown in Figure 2, the whole computational framework could be divided into four steps. First, based on the expression levels of lncRNAs in all the corresponding human tissue or cell types, we calculated tissue specificity scores for the expression of all the lncRNAs. According to a certain empirical cutoff score, we classified these lncRNAs as tissue-specific and non-tissue-specific. We utilized the computational method proposed by Yanai et al. to calculate a tissue specificity score for each lncRNA. The tissue specificity score 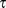 of a given lncRNA is defined as:
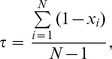



where *N *is the number of tissues, and 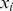 is the expression level of this lncRNA in tissue *i* normalized by maximal expression level in the 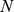 tissues of this lncRNA [45]. In the following steps, we implement different computational processes for these two sets of lncRNAs, aiming to rapidly and accurately predict the diseases associated with them.

Second, for each tissue-specific lncRNA, we first found the corresponding tissue in which this lncRNA had the highest expression level. We considered this lncRNA to be expressed specifically in this tissue; therefore, it might be associated with diseases that are related to this specific tissue. For example, if the tissue specificity score of a certain lncRNA is 0.9, and its highest expression level comes from lung, we inferred that this lncRNA could potentially be associated with lung- related diseases. Although we could not look for a specific lung disease for each lncRNA by this method, it is worth noting that the predicted result has already limited the disease associated with a certain lncRNA to an extraordinarily small area. Here, if two or more tissues simultaneously have the highest expression level, we infer this lncRNA to be associated with these different diseases, the number of which is equal to the number of these tissues.

Third, for each non-tissue-specific lncRNA, we acquire a set of genes co-expressed with this certain lncRNA through computing corresponding Spearman rank correlation coefficients between the integral expression level of this lncRNA and the corresponding genes in the common human tissue or cell types, according to a certain cutoff score.

**Figure 3:**
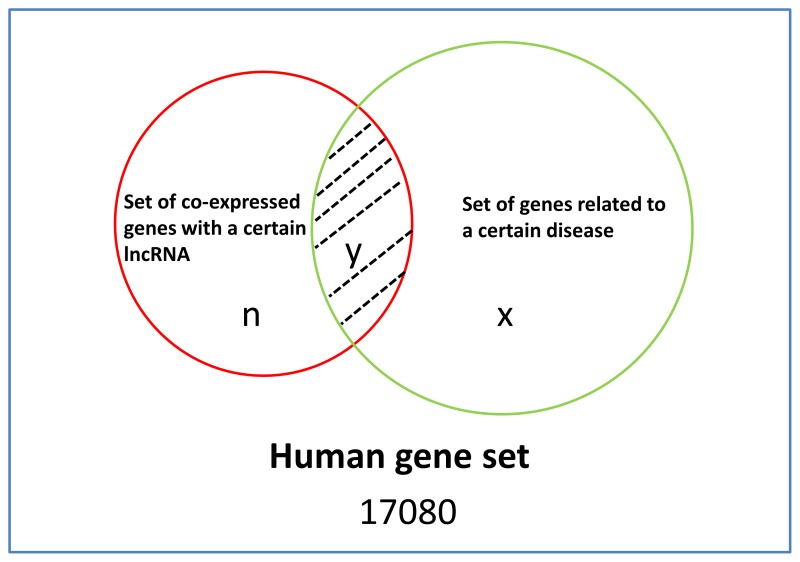
Disease enrichment analysis for the set of genes co-expressed with each non-tissue-specific lncRNA. The blue rectangle represents the whole human gene set, and the corresponding number is 17080. The red circle represents the set of genes co-expressed with a certain lncRNA, and the corresponding number is *n*. The green circle represents the set of genes related to a certain disease, and the corresponding number is *x*. The intersection of these two circles stands for the genes co-expressed with a certain lncRNA and related to a certain disease, and the corresponding number is *y*.

Finally, combining the gene set co-expressed with a given lncRNA with the gene set related to a given disease, the hypergeometric distribution test was then utilized to find significantly enriched diseases in the corresponding co-expressed gene set for each lncRNA. For a certain lncRNA and a certain disease, if 1) the number of co-expressed genes with this lncRNA is *n*, 2) the number of genes related to this disease is *x*, 3) the number of genes which are both co-expressed with this lncRNA and related to this disease is *y*, and 4) the number of genes in the whole human gene set is 17080, then we could calculate a p-value (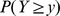), standing for the probability of an event in which the number of genes both co-expressed with this lncRNA and related to this disease *Y* is larger than, or equal to, *y*. The corresponding computational formula is as follows:
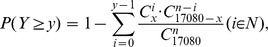



where ‘
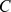
’ is the combinatorial number. The basic idea of the hypergeometric distribution test was shown in Figure 3, where a smaller p-value of hypergeometric distribution test stands for higher enrichment significance. We then used this p-value to measure the enrichment significance of a certain disease in the gene set co-expressed with a certain lncRNA. Based on the corresponding enrichment significance and a certain empirical cutoff score, we could identify potential lncRNA-associated diseases. Ultimately, after performing the above four steps, we could infer the respective disease associated with all the known human lncRNAs.

## Results

### Partition of Tissue-specific and Non-tissue-specific lincRNAs

Based on the above-mentioned method for calculating tissue specificity score, we obtained tissue specificity scores corresponding to 21626 lincRNAs (Table S2). Then we chose 0.8 as a cutoff to distinguish tissue-specific lincRNAs and non-tissue specific lincRNAs. This cutoff has been used similarly in a paper published in 2008 to classify tissue-specific miRNAs and non-tissue-specific ones, in which study Lu et al. performed a comprehensive analysis to the human miRNA-disease association data and dissected some important and interesting patterns among these associations [46]. As a result, we found 13966 tissue-specific lincRNAs and 7660 non-tissue-specific lincRNAs, respectively (Table S2).

### Identification of Potential Associations between Tissue-specific lincRNAs and Disease

Utilizing the above method, we finally found 13720 lincRNAs which have only one specifically expressed tissue, 245 lincRNAs which have two specifically expressed tissues, and 1 lincRNA with three specifically expressed tissues (Table S3). In total, we identified 14213 lincRNA-associated diseases for 13966 tissue-specific lincRNAs (Table S4).

### Recognition of Co-expressed Gene Set for Each Non-tissue-specific lincRNA based on Expression Profiles

For 7660 non-tissue-specific lincRNAs, we first eliminated 1475 lincRNAs which had expression level ‘0’ or ‘1000’ in all 22 human tissue or cell types. Obviously, a lincRNA with an equal expression level of ‘0’ is of no use. We considered lincRNAs with equal expression levels of ‘1000’ in all 22 tissue or cell types to be transcription noise, according to the usual practice in analyzing microarray data. Finally, we got 6185 non-tissue-specific lincRNAs to implement subsequent computational processes.

After that, we calculated Spearman rank correlation coefficient between each non-tissue-specific lincRNA and each gene, based on their expression profiles in 15 common human tissue or cell types. Then we chose 0.7 as a cutoff score aiming to find co-expressed lncRNA-gene pairs. If the Spearman rank correlation coefficient of a pair of lincRNA and gene is larger than or equal to 0.7, we considered them as co-expression. Actually, construction of co-expression networks between genes or RNAs has been implemented by many researchers [47]–[50]. For example, Jordan et al. studied node degree distributions and graphic representations of the corresponding network topologies on different cutoff values, discovering that correlation coefficient 0.7 is an appropriate cutoff [49]. Based on the same principle that Spearman correlation coefficient larger than 0.7, Kuchen et al. 2010 studied co-expression relationship between spliced primary transcripts and mature miRNAs, obtained some conclusions about transcriptional regulation of miRNAs [47]. Some other researchers also achieved meaningful and convictive results utilizing this cutoff value [48], [50]. Thus, according to the experience, we chose 0.7 as a cutoff to construct the corresponding lncRNA-gene co-expression network. By doing so, we obtained a gene set for each lincRNA, containing all the genes co-expressed with this certain lincRNA. As a result, we obtained 602323 co-expressed pairs between 6130 lincRNAs and 15903 genes, with their corresponding Spearman rank correlation coefficients and corresponding p-values.

### Identification of Associations between Non-tissue-specific lincRNAs and Disease

Combining the information about the gene set associated with each human disease, we implemented disease enrichment analysis for the set of genes co-expressed with each non-tissue-specific lincRNA, as described in the Methods. Since our method analyzes multiple disease-related gene sets for the same gene set co-expressed with a certain lincRNA, two classical methods for multiple comparison correction, Bonferroni and FDR, were successively implemented to correct the original p-values. For the final FDR values, we chose 0.05 as a cutoff to select significantly enriched lncRNA-associated diseases. That is, we removed lncRNA-associated diseases with corresponding FDR values larger than 0.05. Thus, we finally obtained 2272 potential lincRNA-associated diseases and their corresponding FDR values. Finally, we reordered these 2272 associations in ascending order according to their FDR values (Table S6). Taking one non-tissue-specific lincRNA, ‘TCONS_12_00025959’, as an example, it was inferred to be associated with Cutaneous T cell lymphoma (CTCL). CTCL is a class of non-Hodgkin’s lymphoma, which is one type of cancer of the immune system. In addition, in each entry of predicted lincRNA-associated diseases, several crucial data are also provided, such as the number of genes that co-expressed with this lincRNA, the number of genes associated with the disease, the number of genes co-expressed with this lincRNA and associated with the disease, and the FDR value. Thus, by combining the known corresponding information about co-expressed lincRNA-gene pairs and gene-disease association pairs (Table S5), researchers could execute several further studies in the future.

The statistical computation in our work was performed in the numerical computing platform ‘MATLAB’ and the statistical platform ‘R’ (Version 2.15.2).

### Performance Evaluation and Result Validation

In order to evaluate our computational framework and validate the predicted results, we chose the LncRNADisease database for reference. This database not only uniquely curates the experimentally supported lncRNA-associated disease data and lncRNAs interaction data at various levels, but also offers a platform that integrates tools for predicting novel lncRNA-associated diseases [15]. At present, LncRNADisease integrates 320 lncRNA-associated diseases between 208 lncRNAs and 166 diseases retrieved from about 500 publications.

We identified some potential relationships between tissue-specific lincRNAs and diseases. However, cross validation could not be implemented on these relationships because we could only predict the potential relationship between tissue-specific lincRNAs and a class of tissue-related diseases other than a specific disease. As a result, we implemented case studies to evaluate the performance of our algorithm. For the top 100 predicted lincRNA-disease associations, we attempted to find evidence through related databases and research articles. Consequently, nineteen of the top 100 associations were verified (Table S7). Especially, four of the top ten predicted lincRNA-disease associations were successfully validated (Table 2). Since lincRNAs and, especially, lincRNA-associated diseases are understudied, this validation result shows good performance of our algorithm. According to the predictive result for tissue-specific lincRNAs, the combinations of lincRNA ‘TCONS_00000720’ and ovarian diseases and lincRNA ‘TCONS_00000721’ and ovarian diseases are separately the first and second most probable associations. The corresponding gene name for these two lincRNAs is ‘EXD3’. In 2011, Yoshihara et al. identified 31 BRCA1-unique CNV regions covering 241 overlapping genes in the samples of BRCA1-associated ovarian cancer patients. The gene ‘EXD3’ is within these 241 overlapping genes, which are associated with some molecular and cellular functions related to ovarian cancer, such as carcinogenesis, cell cycle regulation and apoptosis [51]. The relationship between two lincRNAs, ‘TCONS_00001488’ and ‘TCONS_00000895’, and testicular diseases has also been verified. The corresponding gene name for these two lincRNAs is ‘ZNF502’ and ‘DCAF16’, respectively. An experimental result recorded in the Gene Expression Atlas database showed differential expression of these two genes in cryptorchidism, which is a common testicular disorder [52].

**Table 2:** Case studies to evaluate the performance of our algorithm for tissue-specific lincRNAs. Four of the top ten associations were verified.

LincRNA	Disease	Evidence
TCONS_00000720	Ovary-related diseases	Yoshihara et al., 2011
TCONS_00000721	Ovary-related diseases	Yoshihara et al., 2011
TCONS_l2_00001979	Liver-related diseases	
TCONS_00000360	White blood cell-related diseases	
TCONS_00001767	HLF_r1-related diseases	
TCONS_l2_00002779	Testes-related diseases	
TCONS_00000822	Testes-related diseases	
TCONS_00000077	Brain-related diseases	
TCONS_00000895	Testes-related diseases	Gene Expression Atlas Database
TCONS_00001488	Testes-related diseases	Gene Expression Atlas Database

**Figure 4:**
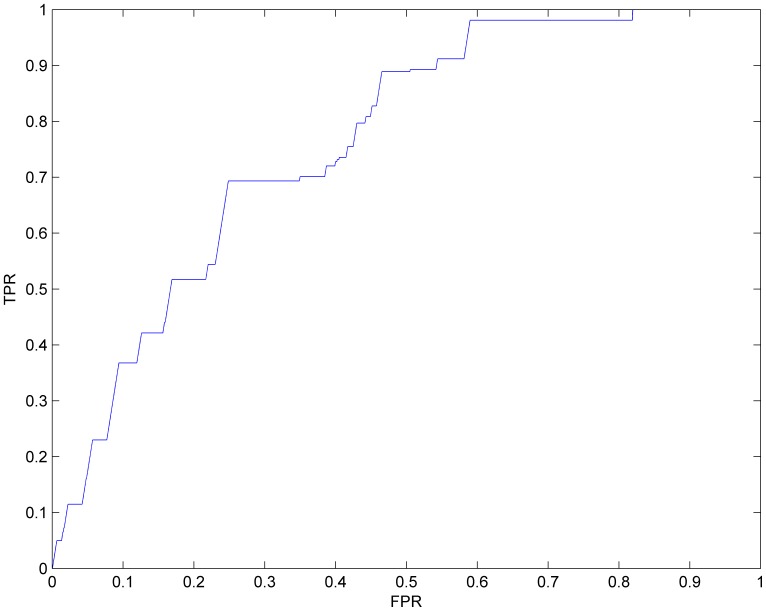
The ROC curve for leave-one-out cross validation and the AUC of our algorithm is 0.7645.

To test the ability of our algorithm to infer potential non-tissue-specific lincRNA-associated diseases, leave-one-out cross validation was implemented on known experimentally verified lncRNA-associated diseases from the LncRNADisease database. Before implementing cross validation, lists of both predictive results and known lncRNA-disease associations were processed in order to confirm that all the lncRNAs and diseases were separately located in the same set. Then we obtained 124 lncRNAs and 19 diseases in total, out of which we finally arrived at 261 known experimentally verified lncRNA-associated diseases and 1338 predictive lncRNA-associated diseases. Subsequently, each known experimentally verified lncRNA-associated disease was left out as test association, while the remaining 260 known lncRNA-associated diseases were taken as seed associations. For each known lncRNA-associated disease, which was left out before, we implemented our algorithm and assessed how well this association ranked in the predictive results. If the rank of this association exceeded a certain given threshold, then the algorithm was considered to have successfully predicted this test lncRNA-associated disease. Finally, the receiver-operating characteristics (ROC) curve was plotted, and the area under the corresponding ROC curve (AUC) was calculated. ROC curve plots true positive rate (sensitivity) versus false positive rate (1-specificity) at different cutoffs. AUC is the area under ROC curve, and AUC = 1 shows perfect performance and 0.5 indicates random performance. As a result, the AUC of our algorithm was 0.7645 (Figure 4). Since our algorithm does not use known lncRNA-associated diseases to make predictions, predictive results exceeding 261 experiments are actually all the same. Thus, the above cross-validation result indicates that our algorithm can recover known experimentally verified lncRNA-associated diseases and, hence, has the potential to identify novel potential lncRNA-associated diseases for non-tissue-specific lncRNAs.

After the leave-one-out cross validation, we then utilized known experimentally verified lncRNA-associated diseases from the LncRNADisease database to validate the predictive results for non-tissue-specific lincRNAs. It should be noted that all the lncRNA-associated diseases we predicted were for lincRNAs, which only represent a part of the whole lncRNAs set. However, entries in LncRNADisease database do not call special attention to lincRNAs, but rather, different kinds of lncRNAs. Therefore, we could only validate part of our predicted results in which the corresponding lincRNAs have now been curated in LncRNADisease database. By doing this, we obtained a subgroup of all the predicted lincRNA-associated diseases for cross validation, which contained 36 associations. As a result, accuracy of prediction showed validation of 32 associations in a total 36 verifiable associations (about 89%) for non-tissue-specific lincRNAs (Table 3).

**Table 3:** Validation of predicted lincRNA-associated diseases for non-tissue-specific lincRNAs, in which 32 of 36 associations have been confirmed by known experimentally verified data in the LncRNADisease database.

LincRNA	Disease	Evidence
TCONS_00017432	Lymphoa, T-Cell, Cutaneous	
TCONS_00015353	Lymphoa, T-Cell, Cutaneous	LncRNADisease verified
TCONS_00015354	Lymphoa, T-Cell, Cutaneous	LncRNADisease verified
TCONS_00014856	Lymphoa, T-Cell, Cutaneous	LncRNADisease verified
TCONS_00015366	Lymphoa, T-Cell, Cutaneous	LncRNADisease verified
TCONS_00015365	Lymphoa, T-Cell, Cutaneous	LncRNADisease verified
TCONS_00015363	Lymphoa, T-Cell, Cutaneous	LncRNADisease verified
TCONS_00015364	Lymphoa, T-Cell, Cutaneous	LncRNADisease verified
TCONS_00015361	Lymphoa, T-Cell, Cutaneous	LncRNADisease verified
TCONS_00015362	Lymphoa, T-Cell, Cutaneous	LncRNADisease verified
TCONS_00015360	Lymphoa, T-Cell, Cutaneous	LncRNADisease verified
TCONS_00015359	Lymphoa, T-Cell, Cutaneous	LncRNADisease verified
TCONS_00014855	Lymphoa, T-Cell, Cutaneous	LncRNADisease verified
TCONS_00014854	Lymphoa, T-Cell, Cutaneous	LncRNADisease verified
TCONS_00017432	Leukemia	
TCONS_00015353	Leukemia	LncRNADisease verified
TCONS_00015354	Leukemia	LncRNADisease verified
TCONS_00014856	Leukemia	LncRNADisease verified
TCONS_00015366	Leukemia	LncRNADisease verified
TCONS_00015365	Leukemia	LncRNADisease verified
TCONS_00015363	Leukemia	LncRNADisease verified
TCONS_00015364	Leukemia	LncRNADisease verified
TCONS_00015361	Leukemia	LncRNADisease verified
TCONS_00015362	Leukemia	LncRNADisease verified
TCONS_00015360	Leukemia	LncRNADisease verified
TCONS_00015359	Leukemia	LncRNADisease verified
TCONS_00014855	Leukemia	LncRNADisease verified
TCONS_00014854	Leukemia	LncRNADisease verified
TCONS_00063838_KCNQ1OT1	Arthritis, Rheumatoid	
TCONS_00017432	Breast Neoplasms	LncRNADisease verified
TCONS_00063838_KCNQ1OT1	Lupus Erythematosus, Systemic	
TCONS_00017432	Carcinoma	LncRNADisease verified
TCONS_00015353	Breast Neoplasms	LncRNADisease verified
TCONS_00015354	Breast Neoplasms	LncRNADisease verified
TCONS_00015353	Carcinoma	LncRNADisease verified
TCONS_00015354	Carcinoma	LncRNADisease verified

The lincRNA-associated diseases in this table are sorted in ascending order of the corresponding adjusted p-value of the hypergeometric distribution test.

## Discussion

As the results show, our method could represent a novel, important and useful resource for lncRNA-associated disease prediction. The innovation of our computational framework could be summarized as follows. First, to the best of our knowledge, it is the first computational method not based on known lncRNA-associated diseases to extensively infer potential human lncRNA-associated diseases, thereby providing a useful supplement to traditional experimental methods. Second, it does not need known experimentally verified lncRNA-associated diseases to identify potential ones, which is particularly important inasmuch as few experimentally verified lncRNA-associated diseases are now known. Third, we established lncRNA-gene co-expressed associations through integrating lncRNAs and gene expression profiles. As a result, we have constructed a computational framework which combines some useful and popular computational methods in the field of gene research with research of lncRNAs, a multiple network platform on which to build more specific computational methods in the future. We utilized a large amount of comprehensive and accurate experimentally verified gene-associated diseases to successfully obtain the potential lncRNA-associated diseases.

It should be noted that our computational framework has two limitations. First, for tissue-specific lncRNAs, our computational framework can only predict their associations with some tissue-related diseases other than more specific disease names. Second, for some human diseases which have few or no related gene records, our method cannot predict their potential associated lncRNAs.

In addition, due to the restriction of available data, the implementation of our computational framework also has some limitations. On the one hand, in this work, we can now only predict potential associations between lincRNAs and diseases as a result of the lack of corresponding lncRNA expression profiles. However, based on our computational framework, more comprehensive results of all classes of lncRNAs could be easily generated as soon as other expression data can be obtained. On the other hand, for lincRNAs, we did not remove redundant ones by comparing their sequence similarity, which might lead to some bias in the final results. However, at present, comprehensive information about lncRNAs is still unavailable. Thus, it is really difficult to obtain sequence information for all the lincRNAs used in our computational process from the current known databases. This limitation could also be avoided as long as comprehensive information about lncRNAs could be obtained.

## Conclusions

Identifying novel potential lncRNA-associated diseases is emerging in bioinformatics as a tool for improving the understanding of disease pathogenesis at the lncRNA level which, in turn, will improve the prognosis, diagnosis, treatment and prevention of human disease. In this work, we proposed the first computational method not based on known lncRNA-associated diseases to identify potential human lncRNA-associated diseases. We first divided all the lincRNAs into two parts: tissue-specific and non-tissue-specific. We quickly and accurately predicted tissue-specific lincRNAs associated with certain tissue-related diseases according to certain criteria. For non-tissue-specific lincRNAs, we first combined lincRNA expression profiles with gene expression profiles to obtain lincRNA-gene co-expressed associations. Then, we implemented a hypergeometric distribution test based on known experimentally verified gene-disease associations to measure the closeness between lincRNAs and diseases. Finally, we evaluated our algorithm and validated our result through case studies and leave-one-out cross validation utilizing the curated LncRNADisease database. The result shows that our method has reliably accurate prediction. Moreover, it is anticipated that with more and more available information about lncRNAs, our computational framework will surely be a useful resource for research on the relationships between lncRNAs and human diseases.

## Supporting Information

Table S1 **Expression profiles of 21626 lincRNAs in 22 human tissue or cell types.** In this table, each row stands for a lincRNA. For a certain lincRNA, several kinds of information have been collected, such as the number of chromosomes in which it locates, its starting position and terminal position in a certain chromosome, its name, and its expression levels in 22 human tissue or cell types.
(XLS)

Table S2 **Tissue specificity scores corresponding to 21626 lincRNAs.** Based on a cutoff score of 0.8, these lincRNAs have been partitioned into 13966 tissue-specific and 7660 non-tissue-specific lincRNAs.
(XLS)

Table S3 **245 lincRNAs which have two specifically expressed tissues and 1 lincRNA which has three specifically expressed tissues.**
(XLS)

Table S4 **14213 predicted potential lincRNA-associated diseases for 13966 tissue-specific lincRNAs.**
(XLS)

Table S5 **51762 associations between 7303 genes and 5150 diseases retrieved from the DisGeNET database.**
(XLS)

Table S6 **2272 predicted potential lincRNA-associated diseases for 6185 non-tissue-specific lincRNAs.**
(XLS)

Table S7 **Case studies to evaluate the performance of our algorithm for tissue-specific lincRNAs.** Nineteen of the top 100 associations were verified.
(XLSX)
